# Experiences of caregivers of community-dwelling older persons with moderate to advanced dementia in adapting the Namaste Care program: a qualitative descriptive study

**DOI:** 10.1186/s40900-022-00401-6

**Published:** 2022-11-12

**Authors:** Marie-Lee Yous, Jenny Ploeg, Sharon Kaasalainen, Carrie McAiney

**Affiliations:** 1 School of Nursing, McMaster University, 1280 Main Street West, Hamilton, ON L8S 4K1 Canada; 2 Schlegel-UW Research Institute for Aging, School of Public Health Sciences, University of Waterloo, 200 University Avenue West, Waterloo, ON N2L 3G1 Canada

**Keywords:** Dementia, Caregivers, Family, Older persons, Workshop, Namaste Care, Community, Qualitative

## Abstract

**Background:**

Globally many older persons with dementia are living at home to maintain independence within the community. As older persons with dementia transition from early to moderate or advanced stages of dementia they require more support from family members and friends to complete their daily activities. Family and friend caregivers, however, often report a lack of preparation for their caregiving role. There are few psychosocial programs that can be delivered by caregivers of community-dwelling older persons with moderate to advanced dementia. Namaste Care is a psychosocial intervention, predominantly used in long-term care, to improve the quality of life of persons with advanced dementia. Namaste Care provides multisensory stimulation for persons with dementia through meaningful activities such as music, massage, aromatherapy, and nutrition. There have been limited attempts at adapting Namaste Care for use by caregivers in the community.There is a need to involve caregivers in adapting programs and understanding their experiences in research involvement so that strategies can be put in place for a positive experience. The purpose of this study is to explore the experiences of caregivers who participated in workshop sessions to adapt Namaste Care for community-dwelling older persons with moderate to advanced dementia.

**Methods:**

A qualitative descriptive design was used. Six caregivers residing in Ontario, Canada attended virtual workshop sessions (i.e., by phone or videoconference) that were guided by the Strategy for Patient-Oriented Research (SPOR) Patient Engagement Framework. Caregivers completed individual post-workshop interviews. Experiential thematic analysis was used to analyze interviews and post-interview researcher notes.

**Results:**

Key findings were that caregivers had a positive experience in adapting Namaste Care by learning how to improve their caregiving skills and being supported to engage in research through multiple facilitators such as flexible scheduling and an inclusive and respectful environment. Having designated time for discussions between caregivers was perceived as important to forming partnerships within the group to support co-creation of knowledge.

**Conclusion:**

Findings support the need to improve caregiver research engagement processes by ensuring that caregivers can benefit through learning opportunities and discussions and empowering caregivers to value their contributions in adapting interventions.

**Supplementary Information:**

The online version contains supplementary material available at 10.1186/s40900-022-00401-6.

## Plain language summary

As older persons with dementia transition from early to moderate or advanced stages of dementia they require more support from family and friend caregivers to accomplish their daily activities. Caregivers, however, often report a lack of preparation for their caregiving role. There are few programs focusing on skill-building and positive interactions that can be delivered by caregivers of older persons with moderate to advanced dementia at home. Namaste Care is a program originally created for long-term care that provides multisensory stimulation for persons with dementia through activities such as music, massage, aromatherapy, and nutrition. It has not yet been adapted for use by caregivers in the community. There is a need to involve caregivers in adapting programs and understanding their experiences in research involvement. The purpose of this study is to explore the experiences of caregivers who participated in workshops to adapt Namaste Care for older persons with moderate to advanced dementia at home. A qualitative descriptive design was used. Six caregivers from Ontario, Canada attended virtual workshop sessions (i.e., by phone or videoconference) and completed individual post-workshop interviews. Interviews and notes were analyzed for themes. Key findings were that caregivers had a positive experience by learning how to improve their caregiving skills and being supported to engage in research through numerous strategies such as flexible scheduling and an inclusive environment. Findings support the need to improve caregiver research engagement by ensuring that caregivers experience benefits from their involvement and empowering them to value their contributions in adapting interventions.

**Supplementary Information:**

The online version contains supplementary material available at 10.1186/s40900-022-00401-6.

## Background

More than 50 million individuals worldwide are affected by dementia [1]. In Canada approximately 60–70% of persons with dementia are living at home to maintain independence and engagement with their community [2, 3]. Close to 50% of Canadians with moderate dementia and 14% of those with advanced dementia live at home instead of a retirement or long-term care home [4]. Family and friend caregivers (hereafter referred to as caregivers) are highly involved in supporting persons with dementia; however, many caregivers report an unmet need related to caregiver education and few persons with dementia are provided with meaningful activities [5]. Meaningful activities for persons with dementia are key to ensure their well-being and enable their participation in daily activities and social relationships [6].

Providing education for caregivers on psychosocial interventions (e.g., sensory activities, cognitive stimulation, exercise, art-based therapy) can provide them with greater confidence to deliver meaningful activities for persons with dementia [7]. Psychosocial interventions can improve the quality of life of both caregivers and persons with dementia by slowing or preventing a decline in mental and physical health of caregivers and/or persons receiving care by targeting caregiver skills, knowledge, activities and/or relationships [8]. These interventions have been found to improve the quality of life of caregivers, their knowledge of dementia, and their competencies in delivering care [9–12].

To date there are few psychosocial approaches suitable for caregivers supporting older persons with moderate to advanced dementia [13]. Most interventions are designed to support caregivers of older persons with early to moderate dementia [9]. Caregivers are often not invited to participate in designing or adapting psychosocial interventions, including those for individuals with moderate to advanced dementia [14–17]. The absence of caregiver voices in intervention development can limit the fit of interventions with their realities of caregiving. Namaste Care is a psychosocial intervention that can potentially be used by caregivers of persons with advanced dementia at home [18], however there have been limited attempts at adapting Namaste Care for use by caregivers in the community, and this area has not been explored in research studies.

Namaste Care offers multisensory stimulation for persons with advanced dementia [18]. Its principles are: (a) providing a comfortable Namaste Care environment and (b) using an unhurried, loving touch approach [18]. In long-term care, Namaste Care is usually delivered in a group setting and begins by welcoming persons with dementia to a quiet room with soft lighting, relaxing music, and soothing scents. They receive person-centred activities including music, massage, socialization, aromatherapy, and nutrition. Namaste Care has gained worldwide recognition and has been used in long-term care, hospice, and acute care settings, and in one study by volunteers in home settings for people at different stages of dementia [19]. The impacts of Namaste Care include the decreased use of anti-anxiety medications and psychotropic medications and reduced pain while improving quality of life for persons with advanced dementia and relationships with others such as staff and family members [20–25]. Only one study implemented Namaste Care in a home setting [19] and family caregivers were not involved in determining how the program should be implemented in a home setting.

Higher quality research is created by involving persons with lived experience to share unique insights regarding their needs that can ultimately lead to the development of solutions to address complex problems [26, 27]. There is more efficient use of research funding when involving persons with lived experience as researchers can ensure that they are asking relevant questions and considering outcomes that are important to service users [28]. People with lived experience include caregivers supporting persons with dementia. Caregivers are valuable partners in collaborating with researchers to develop and refine programs, especially when older persons with more advanced forms of dementia may be unable to collaborate in research [29]. Caregivers have a wealth of knowledge and experience to share with researchers and this valuable information may be missed when they are not provided with opportunities to collaborate on intervention development [30]. Involving caregivers ensures that research is meaningful, results in higher quality research, and eases the way for knowledge transfer [31, 32]. Caregivers may also experience benefits in the process of research collaboration by learning how to engage persons living with dementia in different ways.

Some of the commonly used methods for engaging persons with lived experience (e.g., focus groups, interviews) consist of those often used in qualitative research [33, 34]. Workshop sessions are another type of qualitative method that can be used to involve caregivers in co-producing solutions or generating new ideas to common problems by ensuring that caregivers are provided with opportunities for open discussion [35]. This can help support their comfort in sharing within a new group and promote dialogue. Engagement of persons with lived experience and building trust between researchers and persons with lived experience can be achieved using strategies found within qualitative methods including active listening, reflection, and co-creation of knowledge [36].

Despite the growing trend to involve persons with lived experience in research, few studies explicitly evaluated or explored the benefits or disadvantages for caregivers collaborating in research [37]. There is a need to explore the perceptions of individuals with lived experience in participating in research engagement opportunities. Seeking this feedback can provide insight into how best to support them in research and ensure that they benefit from the experience.

In light of these considerations, the purpose of this study is to explore the experiences of caregivers of community-dwelling older persons with moderate to advanced dementia in adapting the Namaste Care program for home use through workshop sessions. The research questions were: (a) What were the perceived contributions and impacts of caregivers in adapting Namaste Care? and (b) What were caregivers’ perceptions of the factors influencing their experiences in adapting Namaste Care? The current study is part of a multiphase mixed methods study which aims to adapt [38], implement, and evaluate Namaste Care for use by caregivers of community-dwelling older persons with moderate to advanced dementia. The study protocol is published elsewhere [39].

## Methods

### Study design

#### Qualitative description design

The research design for this study consists of qualitative description [40]. This design was chosen because it allows for straight descriptions of phenomena with some flexibility for interpretation [40, 41]. It is important to ensure that the description of caregivers’ experiences in collaborating to adapt Namaste Care reflects their own words. In the next section we provide an overview of the adaptation workshop sessions to shed light on the process of engaging caregivers in adapting Namaste Care.

#### Adaptation workshop sessions

Adaptation of Namaste Care occurred by conducting a total of four small group workshop sessions with caregivers of community-dwelling older persons with moderate to advanced dementia. Each caregiver attended two workshop sessions, an initial workshop session and a follow-up session. Workshops were facilitated by the first author, a Registered Nurse with experience in caring for persons with dementia and their caregivers and conducting caregiver education sessions for the Alzheimer Society. The workshop sessions lasted about 60–90 min. Most caregivers participated by videoconference (i.e., Zoom) except for one who participated by telephone. At the first workshop session, caregivers were provided with a 20-min educational presentation of Namaste Care followed by a detailed discussion on their thoughts and suggestions for adapting the Namaste Care program for use in their homes. Prior to the second workshop session a draft of the Namaste Care training guide based on the first workshop discussion was shared with caregivers by email. The training guide included a full description of the adapted program as well as tips and strategies for caregivers who planned to implement the program. Key topics included a description of the program, steps for delivering the program, examples of sessions, and description of sensorial activities (e.g., music, aromatherapy, range of motion and touch activities). At the follow-up workshop session, the training guide was presented to caregivers followed by a discussion on further revisions required. The content for the workshop sessions were developed based on a review of concepts and processes for delivering the Namaste Care program contained within two books: *The End-of-Life Namaste Care Program for People with Dementia* [18] and *Namaste Care for People Living with Advanced Dementia: A Practical Guide for Carers and Professionals* [42].

The workshop sessions were guided by the Strategy for Patient-Oriented Research (SPOR) Patient Engagement Framework [43]. The term ‘patient’ is a generic term used in this framework and in the context of this study to refer to people with lived experience. Caregivers have lived experience of caring for persons with dementia. The overarching vision of the Patient Engagement framework is to include people with lived experience as active participants in health research to improve health outcomes and lead to a better health system. The SPOR Patient Engagement Framework consists of guiding principles (i.e., inclusiveness, mutual respect, co-building, and support) and targeted outcomes of research engagement [43]. These guiding principles were incorporated before, during, and following the workshop discussions. Inclusiveness was upheld by including diverse caregivers based on sex and relationship with persons with dementia at the workshops and unique caregiving contexts. Mutual respect was achieved by acknowledging the experiences of caregivers in caring for older persons with advanced dementia and using these to draft the training guide. Co-building occurred through collaboration with caregivers in adapting Namaste Care and encouraging caregivers to build on each other’s ideas. Ongoing support was delivered during the adaptation phase by creating a safe and welcoming environment where caregivers felt comfortable in sharing, eliminating the use of technical or medical jargon, and providing education to meet their learning needs. Applying these principles throughout the adaptation process increased the potential for successful engagement of caregivers.

We recognize that our study does not truly reflect co-design as we started with an existing program, so we limited the use of the framework by only incorporating its guiding principles and outcomes. Some of the targeted outcomes of engaging people with lived experience include: (a) inclusive mechanisms and processes created; (b) respectful collaboration between researchers and people with lived experience; and (c) experiential knowledge of people with lived experience being valued as evidence for research [43]. In this study these outcomes were targeted by ensuring that the voices of caregivers were reflected in the adapted Namaste Care program and that the process of developing the approach was a positive experience for caregivers. These perceived outcomes were explored by conducting qualitative interviews with caregivers at the end of the workshop sessions to understand their experiences in adapting Namaste Care. Details regarding the adapted Namaste Care program are available elsewhere [38]. See Additional File 1 for the GRIPP2 reporting checklist [44].

### Setting

Following the adaptation workshop sessions, caregivers met individually with the lead author using a method (i.e., by telephone or Zoom) that was most convenient for them and adhered to the local public health guidelines at the time of the COVID-19 pandemic.

### Sample

A total of six family and friend caregivers of older persons with moderate to advanced dementia, living in urban areas of Ontario, Canada, were included. Caregivers were selected based on purposive sampling, specifically criterion and snowball sampling [45]. Criterion sampling was used to locate participants who were: (a) 18 years and older with experience providing physical, emotional, and/or psychological support at home for a family member or friend aged 65 years or older with moderate to advanced dementia for at least four hours a week within the previous five years; (b) able to speak, write, and understand English; (c) living in Ontario; and (d) capable of providing informed consent. Snowball sampling [45] was used by asking caregivers to share study and researcher contact information with other caregivers who might be interested in participating in the study.

### Recruitment

Caregivers were recruited from the Alzheimer Society of Canada, local Alzheimer Societies, and Dementia Advocacy Canada. The lead author presented the study at virtual Alzheimer Society dementia education series for caregivers (e.g., Care in the Later Stages Series). The study was also presented to clients and staff (e.g., public education coordinators, counsellors, recreational therapists) of the Alzheimer Society. Information about the study was shared through websites, social media and in electronic newsletters of the listed organizations.

### Data collection

Data were collected from October 2020 to January 2021. Demographic data were collected including age, sex, education, relationship to the person with dementia, number of years in the caregiver role, and type of support provided. Individual semi-structured interviews lasting approximately 30–60 min were conducted with caregivers following the completion of the second workshop session. See Table 1 for the interview guide. The interviews explored research engagement experiences of caregivers. Reflective notes were made immediately after interviews. Interviews were audio-recorded and transcribed by an experienced transcriptionist. The interview guide was developed based on a review of the literature for concepts such as patient engagement and workshops, research team expertise, the SPOR patient engagement framework [43], and the Patient and Public Engagement Evaluation Tool [46]. The Patient and Public Engagement Evaluation Tool was developed to assess four principles for effective engagement (i.e., integrity of design and process, influence and impact, participatory culture, and collaboration and common purpose) from different perspectives (e.g., persons with lived experience, members of an organization) [47].

**Table 1:** Interview guide

	Post-workshop interview guide
1	What did you think about the workshop sessions?
2	How did you feel included in discussions? Please explain
3	How comfortable were you in sharing your ideas in adapting the program?
4	How did you feel that your ideas and experiences were being respected during the workshops? Explain?
5	How did you feel that your ideas were reflected in the training guide? Please explain
6	What were the strengths of the workshops?
7	What could have been done differently to make this experience better for you?
8	What would you like us to know about how your participation in the workshops were supported?
9	What would you like us to know about the influence you think the workshops will have?
10	Are there other effects your work in this project had on you?

The interview guide was inspired by the targeted outcomes of the SPOR Patient Engagement framework [42] and the Patient and Public Engagement Evaluation Tool [44]

### Data analysis

Interviews and notes were analyzed using thematic analysis to identify themes and patterns within the data [48]. Experiential thematic analysis was selected as it allows one to focus on the experiences of participants and their perceptions of their world [49]. Braun and Clarke’s [48, 49] method for thematic analysis has been used in other qualitative descriptive studies [50, 51]. Inductive coding helped to identify unique codes that arose from the post-workshop interviews. Deductive coding was also used by analyzing data to fit within a coding framework developed from a review of the literature and the expertise of the research team. We followed the six phases of thematic analysis as outlined by Braun and Clarke [48]: (a) gaining familiarity with the data; (b) conducting coding; (c) locating themes; (d) reviewing themes; (e) developing a definition for themes and naming them; and (f) developing a report.

In gaining familiarity with the data the lead author reviewed the transcripts twice before commencing coding. The lead author analyzed data concurrently as interviews were being completed and sought feedback from the research team regarding the coding process. Constant comparative analysis was used to determine similarities and differences across participants. This approach is consistent with the use of a qualitative description design [40]. Themes were identified from the codes to highlight meaningful patterns within the data. Once themes were identified they were reviewed by all members of the research team for completeness and relevance to the data. We reflected on names for themes and named them by considering fit with the data and appropriateness [48]. We ensured that the story we told reflected the voices of the participants and the data and provided direct quotes of participants to support claims [48]. NVivo version 12 software [52] was used for data management.

### Rigour and trustworthiness

To uphold rigour and trustworthiness in qualitative research, the lead author made notes in a reflexive journal to document personal reactions and former experiences with possible impact on the research process [53]. We implemented strategies to address Lincoln and Guba’s [54] trustworthiness criteria consisting of credibility, transferability, dependability, and confirmability. Credibility was met through investigator triangulation and seeking feedback from members of the research team as they hold expertise in researching and working with caregivers and people with dementia in the community. This process also met the criterion of complementarity and supported the validation of data [54]. Clear descriptions were used to describe the setting and sample of the study to promote transferability of findings [54]. We ensured that the study followed clear logic by conducting a comprehensive review of the current literature to determine gaps in the literature.

### Ethical considerations

Ethics approval was granted from the local Research Ethics Board (#10,526). All caregivers received a written introduction to the study and an informed consent form written in lay language. Caregivers provided oral informed consent to participate in the study. Study IDs were provided for each participant and were used to label data collection forms to maintain anonymity. The three core principles of the Tri-Council Policy Statement, respect for persons, concerns for welfare, and justice, were maintained throughout the conduct of the study [55]. A $25 gift card was offered to caregivers as an incentive.

## Findings

### Demographic characteristics

Characteristics of caregivers involved in adapting Namaste Care were varied in terms of sex, age, highest level of education attained, and number of years in the caregiver role. See Table 2 for the demographic characteristics of caregivers and persons with dementia. Caregivers who identified as being male (*n* = 3) or female (*n* = 3) were equally represented. The mean age of caregivers was 56.5 years, with a standard deviation (SD) of 12.1. Most reported caring for a parent (*n* = 4). All caregivers were actively caring for a family member except for one participant whose parents had died within the previous two years. Most caregivers had completed post-secondary education and half of them had a bachelor’s degree (*n* = 3). More than half were working (*n* = 4). The mean number of years in the caregiver role was 4.9 years (SD = 3.2). All caregivers reported providing support for their family member in the form of advice or emotional support and assistance with daily activities such as maintaining the home, transportation, and managing bills. All except one caregiver reported assisting in personal care activities such as assistance with mobility, meals, and bathing. Older persons with dementia had a mean age of 78.3 years (SD = 7.6).

**Table 2:** Demographic characteristics

Category	*n*
*Caregiver demographics* (*n* = 6)	
*Age in years (Mean [SD])*	56.5 [12.1]
30–49	1
50–59	3
60 and older	2
*Sex*	
Female	3
Male	3
*Ethnicity*	
White/Caucasian	5
Chinese	1
*Highest level of education attainment*	
College diploma	1
Bachelor’s degree	3
Graduate or professional degree	2
*Employment status*	
Retired from paid work	2
Working part-time	3
Working full-time	1
*Relationship to person with dementia*	
Son/daughter	4
Spouse	2
Number of years as a caregiver (Mean [SD])	4.9 [3.2]
1–3	3
4–6	2
7 and up	1
*Person with dementia demographics (n = 7)*	
Age in years (M [SD])	78.3 [7.6]
65–69	1
70–79	2
80 and older	4
Sex	
Female	4
Male	3
*Number of years diagnosed with dementia*	
(Mean [SD])	4.9 [3.3]
1–3	2
4–6	2
7 and up	3

One caregiver was a former caregiver for both parents who were living with dementia and died in the last five years. The age and number of years diagnosed with dementia at the year of their passing were included

### Themes of adaptation experience

Overall, main themes were categorized under: (a) perceived contributions in adapting Namaste Care; (b) factors influencing caregivers’ experiences in adapting Namaste Care; and (c) perceived impacts of learning about Namaste Care. Quotes of caregivers are embedded within the description of themes and identified by a study ID (i.e., CG-X) (Table 3).

**Table 3:** Overview of Namaste Care Adaptation Experience Themes

Categories	Themes
Perceived contributions in adapting Namaste Care	(a) Caregivers felt involved in redesigning Namaste Care
	(b) The training guide reflected caregivers’ ideas and experiences
Factors influencing caregivers’ experiences in adapting Namaste Care	(a) Caregivers felt comfortable sharing ideas for adapting Namaste Care
	(b) Research strategies enabled meaningful engagement of caregivers
	(c) Caregivers appreciated the opportunity to discuss and learn from other caregivers
	(d) Virtual workshops were convenient but limited interactions
Perceived impacts of learning about Namaste Care	(a) Increased awareness of the need for meaningful activities
	(b) Eagerness to start using Namaste Care

#### Perceived contributions in adapting Namaste Care

Caregivers perceived that they made contributions in adapting Namaste Care and in developing the training guide. The themes were: (a) caregivers felt involved in redesigning Namaste Care and (b) the training guide reflected caregivers’ ideas and experiences.

##### Caregivers felt involved in redesigning Namaste Care

Caregivers perceived that they contributed to revising the Namaste Care program so that it could be used in a home setting. They recognized that the purpose of their research engagement was to be actively involved in providing recommendations for adapting Namaste Care. Caregivers felt that not only were their direct suggestions being incorporated in adapting the intervention, but their experiences and situations were being reflected in the recommendations. For example, caregivers felt that it was important to acknowledge that not all activities would work for each person living with dementia.

I appreciate what you are trying to do…hopefully this can help. It’s just hard because every case is different…and that’s when you have to tailor to each individual, but overall you outlined and thought through that as well and these are the different things that can be done. But they won’t all work for everybody. (CG-4)

It was also suggested that caregivers use the adapted program at least twice a week rather than daily as stated in the original Namaste Care program to avoid creating additional burden for caregivers.

##### The training guide reflected caregivers’ ideas and experiences

The training guide that was developed based on workshop discussions with caregivers was perceived as reflecting their ideas and experiences. Caregivers provided various insights in adapting Namaste Care for home use, and this ensured that recommended changes to the program were relevant to their situations. Caregivers were asked to review and comment on the training guide. There was good collaboration between caregivers and the researcher in the process of developing the training guide. Caregivers perceived that the researcher already had knowledge about the program so caregivers felt they had a smaller role in developing the training guide.

I think you knew a lot too to begin with in developing this [training guide]. You weren’t coming out cold turkey. So, you knew a lot already there so I felt that I did part of it. And it was nice to see the stuff that you had. (CG-1)

Some caregivers were therefore not aware that sharing their lived experiences in being a caregiver can also be valuable in informing the development of programs.

#### Factors influencing caregivers’ experiences in adapting Namaste Care

Overall, caregivers reported positive experiences in collaborating to adapt Namaste Care at the workshops. They perceived many factors as supporting their involvement. The themes were: (a) caregivers felt comfortable sharing ideas for adapting Namaste Care; (b) research strategies enabled meaningful engagement of caregivers; (c) caregivers appreciated the opportunity to discuss and learn from other caregivers; and (d) virtual workshops were convenient but limited interactions.

##### Caregivers felt comfortable sharing ideas for adapting Namaste Care

Caregivers reported being comfortable in sharing personal experiences with other caregivers and the lead author. One caregiver stated that she was *“very comfortable”* (CG-4) in sharing. Caregivers perceived that they had enough knowledge of Namaste Care to confidently contribute to adapting the program. They felt included in discussions and that their ideas were respected and valued by all members present at the workshop. Having a small group of people attend each workshop made it easier for all caregivers to share how they envisioned Namaste Care for home use. Caregivers mentioned that a strength within the group consisted of the inclusion of both seasoned and ‘new’ caregivers and varied personalities. *“Because [CG-5] was so forthcoming with his experiences, it made me feel that I need to be more forthcoming of mine too, which wasn’t a problem at all”* (CG-6).

##### Research strategies enabled meaningful engagement of caregivers

Caregivers described a number of research strategies that enabled meaningful engagement such as the creation of a welcoming, respectful safe space to share, flexible scheduling of the workshops, and timely sharing of materials. They reported working on the project to be “*easy*” (CG-3). In terms of resources and scheduling, caregivers reported appreciation for scheduling workshops around their availability. This was especially important for caregivers who were working or required a personal support worker to be present with their family member. *“I think the scheduling and the coordination was done very well”* (CG-5). Caregivers also appreciated receiving materials ahead of time so that they could be prepared for the workshops.

##### Caregivers appreciated the opportunity to discuss and learn from other caregivers

One component of the workshop sessions that was perceived as valuable for caregivers was the opportunity to meet and learn from other caregivers. Caregivers reported learning from the care strategies of others and gained new ideas for activities to try with their own family member. “*Sometimes you live in your own little bubble and you are on your own trying to think of things. So that knowledge and experience from other people has helped”* (CG-4). The workshops took place during the COVID-19 pandemic and many organizations paused in-person caregiver support activities. Caregivers felt that taking part in the workshops allowed them to talk to others about their experiences and challenges.

I think part of it was just a chance to talk to people in different stages of being caregivers and hearing how they are managing it. I think that was a good opportunity. Like any opportunity for caregivers to talk about their experiences and hear other people is always good. I really value that aspect that there’s a little bit of time for debriefing or chatting about what each of us were going through. (CG-2)

Some caregivers reported making new connections with those attending the workshops and wanted to keep in contact.

##### Virtual workshops were convenient but limited interactions

Caregivers perceived that attending workshops by Zoom was convenient as it eliminated the need to travel, arrange for respite care, and the need to book time off work. Caregivers who were working from home felt that it was easy for them to attend the workshops and schedule these around their work hours. Caregivers felt at ease participating as they did not feel rushed and still had time to attend to other activities. The quality of interactions by Zoom was perceived by most as similar to in-person interactions.

I think Zoom actually works very well. It didn’t feel totally different than sitting in a room. When you are in a room, you would notice a little more body language, but I mean you pick up on the facial involvement. And I think that everybody felt comfortable so I actually enjoyed the interaction and the environment, doing it this way. (CG-3)

Although Zoom had its strengths in terms of convenience and simulating face-to-face interactions, it was still perceived by a few caregivers as not as effective as in-person meetings. Some caregivers felt that barriers to meeting virtually were not being able to read the body language of others and being concerned about interrupting others.

At least the way we had Zoom set-up is more of one person goes then the next person goes, the next person goes like that way. What I perceive by losing that is the spontaneity as far as you have an idea that comes to your mind. If I had to hold it for a second that is okay. But if I had to hold it for five minutes, I might forget what I was trying to say. (CG-5)

Caregivers may also not have been aware of how to change Zoom settings to see all attendees at once. One caregiver recommended having a training session on Zoom prior to workshops.

#### Perceived impacts of learning about Namaste Care

Caregivers were learning about Namaste Care for the first time. They had not heard of such a program even though some were caring for a family member for several years. In addition to having the opportunity to contribute to the adaptation of Namaste Care, caregivers benefitted from the experience as they learned about Namaste Care and how they could use this program. The themes related to the impacts of learning about Namaste Care include: (a) increased awareness of the need for meaningful activities and (b) eagerness to start using Namaste Care.

##### Increased awareness of the need for meaningful activities

Although caregivers had implemented some activities in the past, learning about Namaste Care at the workshops made them even more aware of the importance of meaningful activities for older persons with dementia. Caregivers discussed the need to be present and the need for an unhurried approach when interacting with their family member. Namaste Care validated what caregivers were already doing and made them more aware of the need for multisensory stimulation.

Until Namaste Care, I hadn’t really thought a lot about that side of it. I mean there were things we are doing just because, okay it would be nice to do. But, I haven’t really given a lot of thought as to the senses, the five senses that are affected. So yes, it had made me more aware and made me aware that maybe this is something that I have to do. (CG-1)

The workshop sessions provided caregivers with a learning opportunity about how to provide meaningful activities: “*To learn about it [Namaste Care] it’s like taking a very intensive course for two days. Finding out different things that I can do for my mom and going back to my caregiving process. All that was good”* (CG-6).

Caregivers perceived that Namaste Care could help to ensure that their day is filled with activities to keep both caregivers and persons with dementia occupied. Caregivers mentioned how taking part in the workshop sessions had changed their way of thinking regarding how they deliver activities and provided them with more ideas to engage persons with dementia. “*It’s just given me some more ideas. I have been getting limited on what we can do and so it has at least opened my eyes to a few little things that we can do”* (CG-3). Some caregivers mentioned that they would like to share Namaste Care with personal support workers involved in supporting their family member at home.

##### Eagerness to start using Namaste Care

After learning about Namaste Care caregivers wanted to start using it after the workshop sessions. Many caregivers expressed interest in taking part in the next phase of the study which focuses on delivering and evaluating the adapted version of Namaste Care. Some planned to use a chart with dates and times for when to use Namaste Care.

Like with this program…I want to do it three times a week, 20 min a time. I want to put a chart up on my wall and figure out which days I am going to this and what is the timeframe, when I’m going to do it and not when I feel like doing it. (CG-1)

They perceived that the approach was aligned with their usual routines and could be used even after the study is completed.

## Discussion

This is the first study to explore the experiences of caregivers of older persons with moderate to advanced dementia in participating in workshop sessions to adapt Namaste Care for home use. Although one study explored the process of co-designing the Namaste Care intervention for long-term care use with multiple stakeholders [56], the authors did not seek feedback from family members about their experience in co-design workshops. The present study also fills a gap in the current literature related to the involvement of caregivers in adapting or designing interventions to support persons living with moderate to advanced dementia at home. Key findings of the present study included: (a) there were multiple benefits to caregivers in terms of adapting Namaste Care; (b) there is a need to ensure that caregivers recognize their valuable role in research; and (c) numerous strategies contribute to meaningful engagement of caregivers.

While the research team was learning from caregivers in adapting Namaste Care through the workshop sessions, caregivers also benefitted by gaining new knowledge in how to deliver care for family members and the importance of meaningful activities. This finding reveals the extent to which caregivers continue to seek education and support in caring for older persons with dementia at home and how this area remains an important research priority [5, 57]. When caregivers are presented with relevant and timely information in a way that can be easily understood this can positively transform their daily activities of caregiving and improve their confidence in supporting persons with dementia [58].

Despite inviting caregivers to collaborate on adapting a pre-selected intervention in the present study, there were multiple impacts on the project and caregivers themselves. Caregivers perceived that they made valuable contributions in adapting Namaste Care and learned about how they could use Namaste Care to implement meaningful activities at home. Findings from the study reveal the need to empower caregivers in their role in research so that they recognize the value of their research involvement. There are many roles that people with lived experience can play in research. As seen in the present study some roles involve adapting certain parts of research such as interventions through co-development or sharing experiences, ideas, and preferences to help inform the decisions of the research team [59]. Adaptations made to Namaste Care enabled the program to reflect the realities of caring for persons with moderate to advanced dementia living at home. There is a need for more interventions that can be used by caregivers supporting this population [13]. Future studies may want to engage caregivers even more by involving them early on in deciding about research questions and selecting interventions [60]. Although direct quotes of participants were included in this study to support findings, this paper was written from the perspective of the research team. There is a need to increase the voices of caregivers of persons with dementia by involving them in study outputs such as writing manuscripts about their experiences in research [60].

The present study revealed the importance of using several strategies to support the engagement of caregivers in adapting Namaste Care. These strategies can be used to mitigate barriers in research engagement such as lack of perceived direct benefit and distrust of researchers [61]. Recognizing that most caregivers who attended workshops were actively caring for older persons with moderate to advanced dementia, strategies were put in place to support their engagement including flexible scheduling, offering respite care, and creating a welcoming and inclusive environment for sharing. These strategies were aligned with the guiding principles (i.e., inclusiveness, mutual respect, co-building, and support) of the SPOR Patient Engagement Framework and targeted outcomes of research engagement [43]. Opportunities for research engagement should be scheduled conveniently for caregivers and web-based videoconferencing platforms (e.g., Zoom) can be used as an alternative to in-person meetings [61]. As a result of these strategies caregivers perceived that they contributed to the development of the adapted Namaste Care program and training guide and were well supported to participate in research. Caregivers perceived participating in research to be a positive experience and they benefitted from learning about Namaste Care. This indicates the potential for caregivers to participate in similar research in the future.

A key factor in engaging in research mentioned by most caregivers was having time to discuss and share experiences with other caregivers at the workshops. Using an authentic partnership approach when collaborating with people with lived experience in finding solutions to improve and better understand dementia care provides opportunities for shared learning, dialogue, and critical reflection [62]. In the present study caregivers appreciated learning from each other’s experiences to inform their own caregiving processes. In another study family members perceived that having more time for discussion was considered essential in being able to form partnerships [60]. Spending time to get to know each other, including the researcher, was important in helping caregivers feel comfortable in sharing personal stories.

In the present study we involved caregivers in adapting Namaste Care and this strategy has multiple advantages when implementing the intervention in the real-word context. Adaptations made to interventions through engagement of people with lived experience have been found to lead to less burden for those delivering the intervention, greater fit between interventions and preferences, and increased intervention adherence [59]. Caregivers appreciated participating virtually in adapting Namaste Care as this eliminated the need for travelling, taking time off work, and minimized disruptions when caregiving at home. To date few studies included virtual workshops to adapt programs for persons living with dementia. One project conducted during COVID-19 that involved the engagement of a stakeholder advisory council for research in dementia care similarly found that virtual meetings using Zoom technology had benefits for clinicians, caregivers, and persons with dementia such as eliminating the need for travel and creating an enjoyable experience [63].

### Strengths and limitations

This study provided a comprehensive description of the perceived contributions, impacts, and factors influencing the experiences of caregivers in adapting Namaste Care. All except one caregiver who participated in the study were active caregivers. The former caregiver was last caring for a family member two years previously and still strongly identified with the caregiver role [64]. Former caregivers may feel a need to seek opportunities to help other caregivers of people with dementia by sharing their experiences and solutions [65, 66]. Although a small sample size was included in the study, caregiver characteristics varied in terms of sex, relationship to the person with dementia, and length of time as a caregiver. With regards to the SPOR Patient Engagement framework [43], this study included a small sample of lived experiences and future adaptations may be needed to address additional varied experiences of other family caregivers.

A lack of cultural diversity among caregivers was a limitation as all but one caregiver identified as being White/Caucasian. This limits the transferability of the findings particularly for multi-cultural countries such as Canada as people of different cultures may not be as receptive to certain activities proposed in the Namaste Care program such as receiving touch by others. Future work should involve tailoring the program to various cultures. There is a need to target culturally diverse caregivers as Black, Hispanic, Asian and Indigenous Peoples have been found to face discrimination in seeking dementia care and participating in research [67].In this study the group was fairly homogenous with regards to culture and ethnicity. This may have impacted how comfortable participants were in sharing personal stories. Future studies should include a more diverse group and may need to consider differences in ease in sharing personal stories in relation to diverging norms around content discussed, communication styles and preferences regarding discussion format.

We also did not include older persons with dementia in adapting Namaste Care. Engaging both persons with dementia and caregivers at the same time may however impact their comfort level for full expression [68]. Future studies should involve persons living with dementia in the design of the Namaste Care program and other interventions so that research is driven by their needs and preferences. Caregivers were not involved in the research design. Their roles were limited to designing the adapted Namaste Care program and the training guide. Future research should involve caregivers early on so that they can contribute to informing the research design. They should be involved in various research processes such as developing research questions, selecting the study design, establishing study outcomes, interpreting study results, and identifying study implications. In this study caregivers may have been limited by the time available to speak during workshop sessions. There is a need for researchers to offer more opportunities for caregivers to provide feedback in future studies such as provision of written feedback.

Another limitation consisted of possible respondent bias introduced in having the lead author conduct both the workshops and interviews as time and resources did not permit the hiring of an external member to conduct these. There were some advantages as well with regards to helping participants feel comfortable in disclosing their experiences due to prior relationships formed at the workshop sessions. Participants were comfortable in sharing their thoughts including areas for improvement in relation to the use of virtual technology for workshops. In future research, the interventionist and interviewer should be different persons. Galdas (2017) states that qualitative researchers consist of a central part of the research process, and separating researchers from the process is not possible nor desirable [69]. The concern should be focused on transparency and reflexivity with regards to how data were collected, analyzed, and reported [69], which were considered throughout the study as discussed in the methods.

## Conclusion

Overall, caregivers perceived that they had a positive experience in collaborating through workshop sessions to adapt the Namaste Care approach to be used by caregivers of older persons with moderate to advanced dementia in their homes. They enjoyed opportunities for rich learning and to be able to contribute their experiences and ideas in adapting Namaste Care. Findings reveal the need to recognize that caregivers can benefit from education in participating in research and the need to empower caregivers to value their contributions in developing interventions. Implications of the findings are to better integrate caregiver engagement in research by seeking feedback from these individuals regarding their experiences. Caregivers need to be supported to engage in research through multiple strategies. The next step for this study is to support caregivers in using the adapted Namaste Care program and evaluate its implementation and preliminary effects.

## Supplementary Information

**Additional file 1.** GRIPP2 reporting checklist.

## Data Availability

The data for this research consists of questionnaires, interviews and transcriptions and notes. Raw data cannot be publicly released due to the risk of compromising participant confidentiality.

## Abbreviation

**SPOR**: Strategy for patient-oriented research
