# Supplementary material for: Experiences of caregivers of community-dwelling older persons with moderate to advanced dementia in adapting the Namaste Care program: a qualitative descriptive study
Source: Res Involv Engagem. 2022 Nov 12;8:61. doi: 10.1186/s40900-022-00401-6 (PMC9655803; doi:10.1186/s40900-022-00401-6)
Supplement: Supplementary file 1 — Additional file 1. GRIPP2 reporting checklist. [file 40900_2022_401_MOESM1_ESM.docx]

**Additional File 1**

**Table 1. Guidance for Reporting Involvement of Patients and the Public (GRIPP2)-Short form**

| **Section and topic** | **Item** | **Reported on page No** |
| --- | --- | --- |
| 1. Aim | Report the aim of PPI in the study. | Page 8 |
| 2. Methods | Provide a clear description of the methods used for PPI in the study. | Pages 8 to 14 |
| 3. Study results | Report the results of PPI in the study, including both  positive and negative outcomes. | Pages 14 to 20 |
| 4. Discussion and conclusions | Comment on the extent to which PPI influenced the study overall. Describe positive and negative effects. | Pages 21 to 26 |
| 5. Reflections/critical perspective | Comment critically on the study, reflecting on the things that went well  and those that did not, so others can learn from this experience. | Pages 24-26 |

PPI=Patient and public involvement

*Note.* The checklist was taken from: Staniszewska S, Brett J, Simera I, Seers K, Mockford C, Goodlad S, et al. GRIPP2 reporting checklists: tools to improve reporting of patient and public involvement in research. Res Involv Engagement. 2017 Aug 2;3(13).
